# Hand-assisted Endoscopic Surgery: Lights and Shadows

**DOI:** 10.4103/1319-3767.74428

**Published:** 2011

**Authors:** Eduardo M. Targarona

**Affiliations:** Director of the Unit of Gastrointestinal and Hematologic Surgery, Hospital de la Santa Creu I Sant Pau, Professor of Surgery, Autonomous University of Barcelona, Barcelona, Spain

Minimally invasive surgery (MIS) represents one of the fastest and most critical advances in general surgery over the last three decades. It has not only changed the route of abdominal access, but also some clinical paradigms (early discharge, quick refeeding) and the treatment algorithm of a number of surgical diseases (gastroesophageal reflux disease, achalasia, bile duct stones). At the same time, it has stimulated surgical imagination and inventiveness, generating ideas and new tools such as the harmonic scalpel, NOTES and single port access. However, MIS is significantly more difficult to master and perform than conventional open surgery. It is for this reason that the spread and dissemination of some indications for MIS are extremely slow, despite evidence-based findings that they have clinical advantages over their open counterpart (i.e. colorectal surgery). The main difficulty with the performance of MIS is the lack of depth in a two-dimension (2D) screen and the loss of tactile feeling. A variety of attempts have been made to solve these inconveniences in several ways: i) 3D systems, ii) hand-assisted surgery and iii) robotic surgery. Three-dimensional systems have not as yet proven to be superior to conventional endoscopic surgery, as we await newer systems after the ‘avatar 3D’ fashion applied on clinical medicine. Robotic surgery overcomes some of the limitations of conventional MIS. However, its cost and the lack of evidence of superiority over conventional MIS have limited its use to just a few indications. Furthermore, its success appears to be more related to marketing and non-academic reasons than to well-defined scientific knowledge.

A few years after a laparoscopic cholecystectomy was described for the first time, several surgeons proposed hand-assisted laparoscopic surgery (HALS).[1,2] The concept and rationale of HALS is obvious. An air-tight seal allows the hand to be introduced inside the abdomen while maintaining the pneumoperitoneum and recovering tactile feeling. This hybrid approach could overcome the main drawbacks of MIS while possibly maintaining some of its advantages. However, HALS has never been seriously considered as a viable option to MIS or to open surgery. Its main criticism comes from purist endoscopic surgeons and is based on the rationale that this is not MIS, and that HALS should probably only be considered by unskilled endoscopic surgeons. Other surgeons have conducted more scientific approaches to determine whether HALS truly offers advantages for MIS. In this issue of the Journal, Mishkhes *et al*. present their initial experience with HALS in 25 patients, with an overall complication rate of 24% and mortality of 4%.[3]

To analyze the usefulness of HALS we need to divide this topic into several parts: devices, indications, evidence-based medicine and clinical use.

Devices for HALS have undertaken a journey from skin-adherent platforms and glove-based devices to current instrumentation, with iris-based devices that are easy to implant and handle. Good examples are the GelPort (Applied) and the Dextrus (Ethicon) that are reliable and safe.[4]

Clinical experience was initially applied to all abdominal operations, including colorectal, esophagogastric, spleen and obesity surgery. Logical thinking about the use of HALS was to employ this in the case of laparoscopic-assisted surgery, meaning those cases needing an accessory incision. A paradigmatic indication is colorectal surgery because a minilaparotomy is required to retrieve the specimen and to prepare the anastomosis. Another clearly useful indication, and maybe, the most accepted to date is management of an enlarged solid organ, as is the spleen in cases of splenomegaly. In this situation, the intraabdominal hand permits to expose the operating field. Manipulation and injury from endo-retractors can thus be avoided and the accessory incision could be used to extract the whole organ in a clinical situation in which the introduction of an enlarged spleen could be especially time consuming and frustrating.

However, once we have the devices and potential indications, we then need precise knowledge about the clinical features of HALS as compared to open or conventional MIS procedures. There is a lack of evidence-based data on this topic, but information available is consistent. We analyzed the outcome of HALS in a prospective randomized trial, and we found that the clinical outcome was exactly the same and that HALS avoided conversion to open surgery in a percentage of 17%. We also analyzed the inflammatory response because it can be argued that in conventional MIS colorectal procedures, the accessory incision is performed at the end of the procedure, while during HALS, the incision is made at the beginning and stretched throughout the entire surgical process. Through the analysis of IL6 and PCR, we confirmed that HALS was more invasive in terms of tissue injury than conventional surgery, but it did not have any clinical impact on the immediate outcome.[5] A recent meta-analysis showed a similar outcome.[6]

Evidenced-based data on the advantages of HALS in cases of splenomegaly are scarce. In a non-randomized comparative study we showed the definitive advantages in terms of operative time, blood loss, conversion and clinical outcome when HALS was used in cases of spleen with a final weight superior to 800 g.[7]

Other surgeons have highlighted the potential use of HALS in other complex laparoscopic resections. Clear advantages of HALS have also been seen in urological surgery, especially for living donor kidney retrieval[8] or hepatic surgery.[9]

Finally, what exactly could be the final role of HALS? From my point of view, HALS should be considered an alternative technique with different strategies than conventional pure endoscopic surgery, and there are three scenarios for its applications.

1. HALS in case of splenomegaly or in any situations during this type of surgery before the conversion.
2. As a last resort before conversion to manage intraoperative situations during colorectal surgery. The surgeon should keep this possibility in mind and think carefully about the placement of the device for the best management of the intraabdominal difficulty (large tumor, adhesions, long colon loop etc).
3. HALS may be a good alternative to conventional pure endoscopic surgery when the case load is too low to maintain or sustain the learning curve for conventional pure endoscopic colorectal surgery. However, its advantages as a bridge are as yet not convincing, because the maneuvers and skills during HALS are completely different from those of pure endoscopic surgery.

HALS is not pure endoscopic surgery, the technique is not similar and the aesthetic disadvantage is clear. However, it is a clear concept, with accepted indications and good outcomes. It deserves its place in the surgical armamentarium. Judicious use of HALS could help to solve critical situations, and it can enable the advantages of MIS in an environment where pure MIS is difficult to apply due to a short caseload or high technical difficulty.
